# MRC-5 Cancer-associated Fibroblasts Influence Production of Cancer Stem Cell Markers and Inflammation-associated Cell Surface Molecules, in Liver Cancer Cell Lines

**DOI:** 10.7150/ijms.34758

**Published:** 2019-08-06

**Authors:** Song-Ming Ding, Jian-Fang Lu, Muhammad Ibrahim Alhadi Edoo, Lin Zhou, Hai-Yang Xie, Shu-Sen Zheng, Qi-Yong Li

**Affiliations:** 1 Shulan (Hangzhou) Hospital, Hangzhou, Zhejiang, P.R. China; 2 Key Laboratory of Combined Multi-organ Transplantation, Ministry of Public Health; Key Laboratory of Organ Trans-plantation, Zhejiang Province; Hangzhou, Zhejiang, China; 3 First Affiliated Hospital, Zhejiang University School of Medicine, Hangzhou, Zhejiang, P.R. China

**Keywords:** cancer-associated fibroblast, cancer stem cells (CSCs), pro-tumoral inflammation molecules, cancer progression

## Abstract

**Background:** Current opinion suggests that expansion of cancer stem cells (CSCs) and activation of pro-tumoral inflammation cascade correlate with cancer progression.

**Materials and methods:** We explored the possible contributions of MRC-5 cancer-associated fibroblasts to the expression profiles of CSC markers and inflammation-associated cell surface molecules. The liver cancer cell lines Bel-7402, SMMC-7721, MHCC-LM3, and HepG2 cultured in conditioned medium (CM) from MRC-5 served as test groups, whereas the liver cancer cell lines cultured in normal medium served as control groups.

**Results:** Flow cytometry revealed that the proportions of CD90^+^ cells were significantly higher in MHCC-LM3-(MRC-5)-CM and HepG2-(MRC-5)-CM cells, and moderately higher in Bel-7402-(MRC-5)-CM and SMMC-7721-(MRC-5)-CM cells, than in controls. The CD90^+^/CD45^-^ proportions were elevated in Bel-7402-(MRC-5)-CM and MHCC-LM3-(MRC-5)-CM cells, but reduced in HepG2-(MRC-5)-CM and SMMC-7721-(MRC-5)-CM cells, as compared to controls. Western blotting indicated that Nanog was downregulated in MHCC-LM3-(MRC-5)-CM and HepG2-(MRC-5)-CM cells, compared to controls; that POU5F1 (OCT4/3) was downregulated in MHCC-LM3-(MRC-5)-CM, but upregulated in Bel-7402-(MRC-5)-CM and HepG2-(MRC-5)-CM cells, compared to controls, and that CK19 was upregulated in Bel-7402-(MRC-5)-CM and MHCC-LM3-(MRC-5)-CM cells, compared to controls. Proportions of cells expressing Toll-like receptor-1^+^ (TLR1) and TLR4 were significantly higher in MHCC-LM3-(MRC-5)-CM cells, and moderately higher in HepG2-(MRC-5)-CM cells, than controls. However, the TLR1^+^ and TLR4^+^ proportions were lower in Bel-7402-(MRC-5)-CM and SMMC-7721-(MRC-5)-CM cells than controls. Proportions of CD25^+^ cells were reduced in HepG2-(MRC-5)-CM and SMMC-7721-(MRC-5)-CM cells, but elevated in MHCC-LM3-(MRC-5)-CM and Bel-7402-(MRC-5)-CM cells, compared to controls. Proportion of CD61^+^ cells was higher in liver cancer cells cultured in MRC-5-CM than in controls. Proportion of CD14^+^ cells was lower in HCC cells cultured in MRC-5-CM than in controls.

**Conclusion:** MRC-5 extensively affected the production of CSC markers and inflammation-associated cell surface molecules. Tumor-targeting molecular therapies should consider these findings.

## Introduction

Liver cancer is one of the most common malignancies worldwide 1. Although great advances in surgery and treatment have been made over the past decades, the prognosis remains pessimistic. The high-level motility and aggressiveness of liver cancer cells cause therapies to fail.

The current hypotheses on how liver cancer evolves may be termed as the tumor microenvironment (TME) 2, epithelial-to-mesenchymal transition (EMT) 3, and the cancer stem cell (CSC) hypotheses 4. The TME is a complex mixture of cancer cells, endothelial cells, immune cells, fibroblasts, cancer-associated fibroblasts (CAFs), the extracellular matrix (ECM), and soluble factors 5. The TME is a critical regulator of both cancer progression and distant metastasis. Three-quarters of all liver cancer are attributable to chronic infections with hepatitis B and C viruses 6. Thus, the TME of liver cancer is distinctive because it includes hepatitis-virus-associated inflammatory cytokines and chemokines. Tumor-associated macrophages (especially M2 macrophages) play pivotal roles in liver cancer initiation and progression 7. M2-type macrophages exert multiple functions; activating T helper 2 (Th2) cells, facilitating escape from immune surveillance, promoting proliferation of tumor cells, and inducing angiogenesis. M2-type macrophages may be distinguished from peripheral blood monocytes upon exposure to toll-like receptor (TLR) ligands. TLRs, an important family of pattern-recognition receptors, are highly expressed in immune cells. Notably, TLRs can also be expressed by hepatocytes, stellate cells, and liver cancer cells 8. However, any role for TLRs in liver cancer evolution remains to be elucidated; any association of TLRs with liver cancer immune escape remains elusive.

EMT is intimately implicated in cancer progression and metastasis 9. EMT is both phased and reversible. Cancer cells undergoing EMT acquire mesenchymal properties (a spindle-like shape with enhanced invasion and migration potentials), and experience loss of cell-cell adhesion (mainly because of disruption of E-cadherin/catenin complexes on the cell membrane or downregulation of epithelial marker expression). Emerging evidence supports the idea that liver cancer is orchestrated by CSCs, a rare population of cells with the ability to self-renew and form mammospheres 4, 8. CSCs are responsible for carcinogenesis and tumor progression 10, 11. Identification of “global consensus” markers for CSCs is critical to combat malignant tumors. To date, several potential biomarkers have been investigated; these include CD24, CD44, CD90, CD45, CK7, CK19, POU5F1, Nanog, and Sox2 12-14. Also, several signaling pathways have been suggested to play roles in CSC differentiation; these include the Wnt, TGFB1/CTNNB1, Jagged1/Notch, Hedgehog, IL-6/stat3, and HGF/MET pathways 7.

EMT is associated with the generation of cancer cells that exhibit stem-cell-like characteristics. Furthermore, many authors have reported that CAFs can initiate EMT 15. CAFs are major components of stromal cells, exhibiting upregulated expression of skeletal muscle alpha-actin. CAFs originate from fibroblasts, myofibroblasts, and endothelial cells. The lung is rich in fibroblasts, and liver cancer cells tend to spread to the lung 16. Therefore, we have evaluated the effect of MRC-5-CM on the expression of CSC markers and inflammation-associated cell surface molecules to elucidate the growth of, and metastatic tumor formation by, liver cancer.

## Materials and methods

### Cell culture

The human lung fibroblast cell line MRC-5 was a generous gift from Dr. Xi, Chen (Zhejiang University, China). Bel-7402, SMMC-7721, MHCC-LM3 and HepG2 cell lines were purchased from Shanghai Cell Bank, Chinese Academy of Sciences. MRC-5 cells were maintained in Dulbecco's modified Eagle's medium (DMEM) (Gibco, Grand Island, NY, USA) supplemented with 10% heat-inactivated fetal bovine serum (Sigma-Aldrich, St. Louis, MO, USA) at 37°C in a 5% CO2 water-saturated environment. Conditioned medium of MRC-5 cells (MRC-5-CM) was collected as follows: cells were cultured until 70-90% confluency, at which point the used medium was collected and passed through a 0.22-μm filter, diluted at a 1:1 ratio with DMEM containing 10% FBS. DMEM medium supplemented with 10% FBS served as the control medium. Bel-7402, SMMC-7721, MHCC-LM3 and HepG2 cells were respectively cultured in the MRC-5-CM for 14 days (n=3). MRC-5 and MHCC-LM3 were subcultured once a week at a ratio of 1:1. 5ml MRC-5-CM was used when liver cancer cells were cultured in 25cm2 cell culture flasks. 20ml MRC-5-CM was used when liver cancer cells were cultured in 75cm2 cell culture flasks.

### Western-blot Analysis

Following culture in MRC-5-CM for 14 days, whole liver cancer cells were lysed on ice in a lysis buffer (RIPA, Beyotime, Shanghai, China) with a protease inhibitor mixture cocktail (Roche, Switzerland). After centrifugation at 12000rpm for 30 minutes at 4℃, the protein concentrations of supernatants in samples were measured by the BCA protein assay (Thermo scientific, Rockford, IL, USA). Equal amounts of protein (50μg) were separated by 10%-12% NUPAGE Bis-tris Gel (Invitrogen, CA, USA) electrophoresis (constant voltage: 120mv) and transferred onto polyvinylidene fluoride (PVDF, 0.45μm) membranes (constant current: 350mA for 70/120 min). After being blocked by Tris-buffered saline and Tween 20 (TBST) buffer containing 5% non-fat powder milk for 2h, the membranes were incubated with primary antibodies overnight on ice. After washing the membranes several times in TBST while agitating, detection was performed using the appropriate secondary HRP-conjugated anti-mouse or anti-rabbit antibody. Immunoreactive bands on the blots were visualized with enhanced chemiluminescence reagent ECL kit (Beit Haemek, Israel). Anti-GAPDH, anti-WNT-2, anti-WNT-5B, anti-WNT16, anti-TGFB1, anti-CTNNB1, anti-IL6, anti-Nanog, anti-OCT4 and anti-CK19 primary antibodies were purchased from (Epitomics).

### Confocal immunofluorescent analysis

The 5× 105 cells were implanted onto a cell culture dish for 24 hours (NEST Biotech, Hong Kong, China) after culturing in MRC-5-CM for 14 days. Cells were fixed with paraformaldehyde for 30 minutes, then permeabilized with 0.1% Triton X-100 for 10 minutes at room temperature, and thereafter sealed with goat serum for 1 hour at room temperature following primary antibodies incubation in the dark for 24 hours at 4°C. Washed three times with PBS, the cells were then incubated with Alexa Flour® 488 IgG donkey anti-mouse or anti-rabbit second antibodies (1:300, Invitrogen, USA) in the dark for 1 hour at room temperature. Fluorescence images were photographed with confocal microscopy (Leica DMIRE2, Germany) (at 10×63 magnification).

### Flow cytometry

The presence of CD14, CD25, CD28, CD45, CD61, CD90, TLR1 and TLR4 were analyzed using a flow cytometer (CYTOMICS FC 500, Beckman Coulter, Miami, FL) according to manufacturer's instruction. Anti-CD14-PE-Cy7, CD25-FITC, TLR4-PE, CD28-PE, CD61-PE, CD90-FITC and CD45-APC were purchased from (BD Biosciences) ; anti-TLR1-PE was purchased from (eBioscience).

### Colony formation assay

The 1× 103 cells were allowed plating in an 8 cm plate. After two weeks of culture, the colonies (>10cells) were stained with crystal violet and counted.

### Statistical Analysis

Student's t-test was performed to compare the differences between the 2 groups using SPSS 16.0 software (SPSS, Chicago, IL, USA). Statistical significance was set to p<0.05.

## Results

### Effects of MRC-5-CM on expression of CSC markers and associated signaling pathways

**Figure 1:**
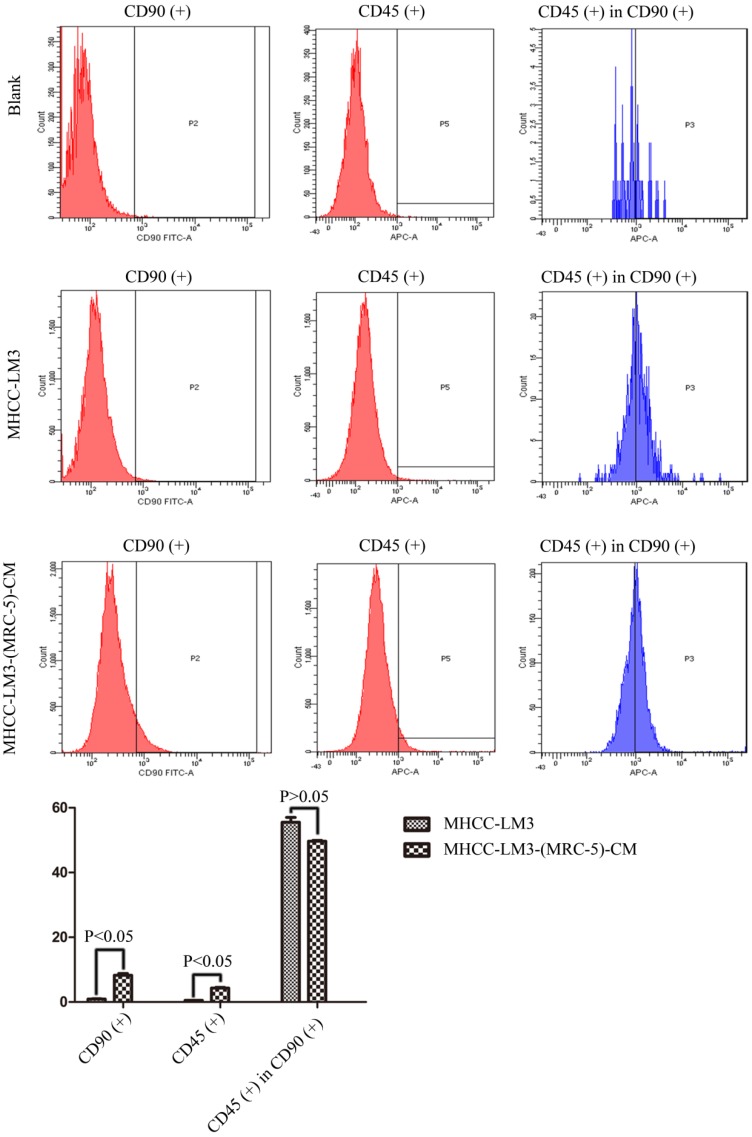
The CD90+ and CD90+/CD45- populations of MHCC-LM3-(MRC-5)-CM cells compared to those of MHCC-LM3 cells.

**Figure 2:**
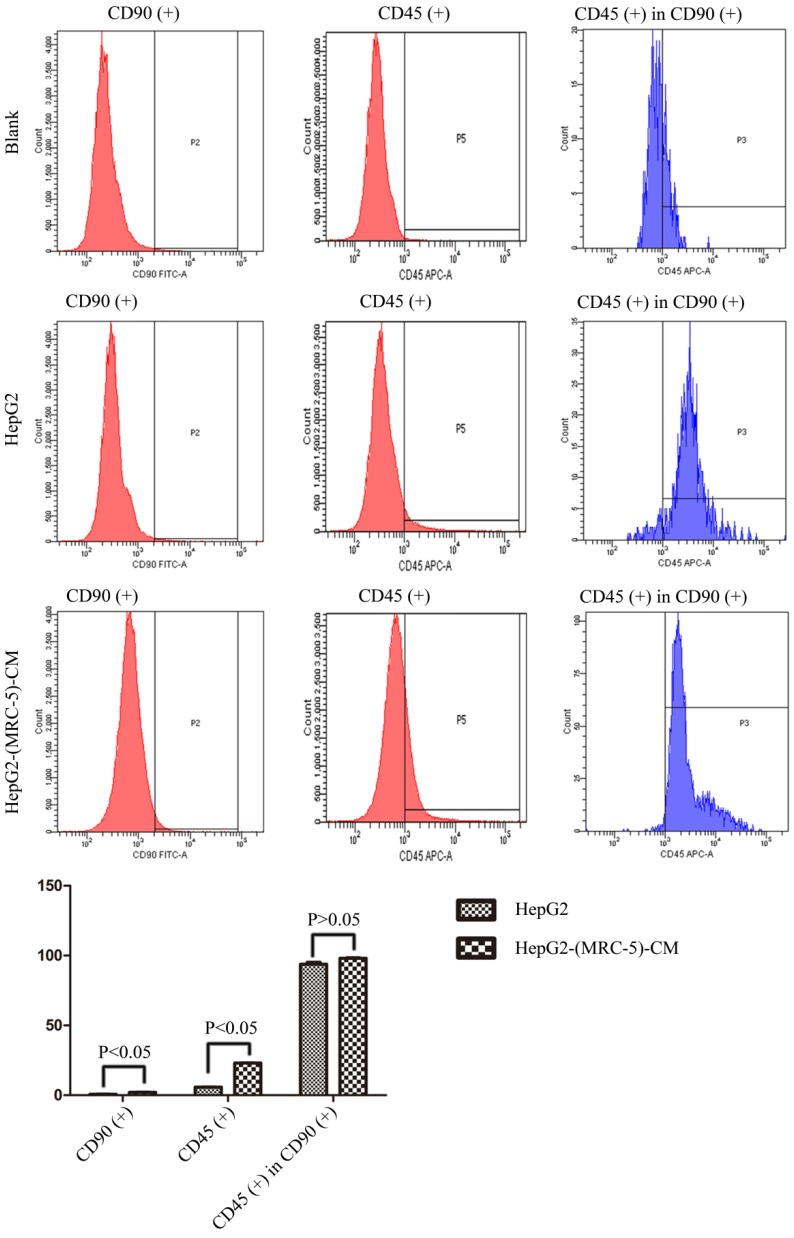
The CD90+ and CD90+/CD45- populations of HepG2-(MRC-5)-CM cells compared to those of HepG2 cells.

**Figure 3:**
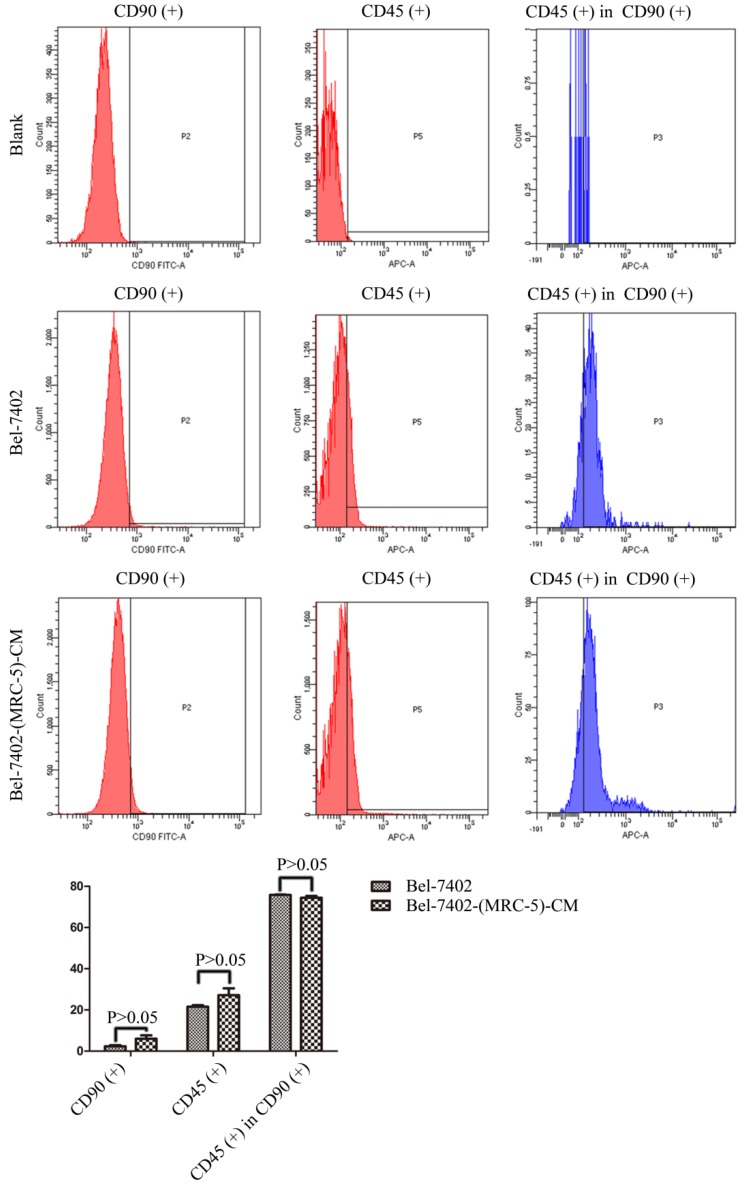
The CD90+ and CD90+/CD45- populations of Bel-7402-(MRC-5)-CM cells compared to those of Bel-7402 cells.

**Figure 4:**
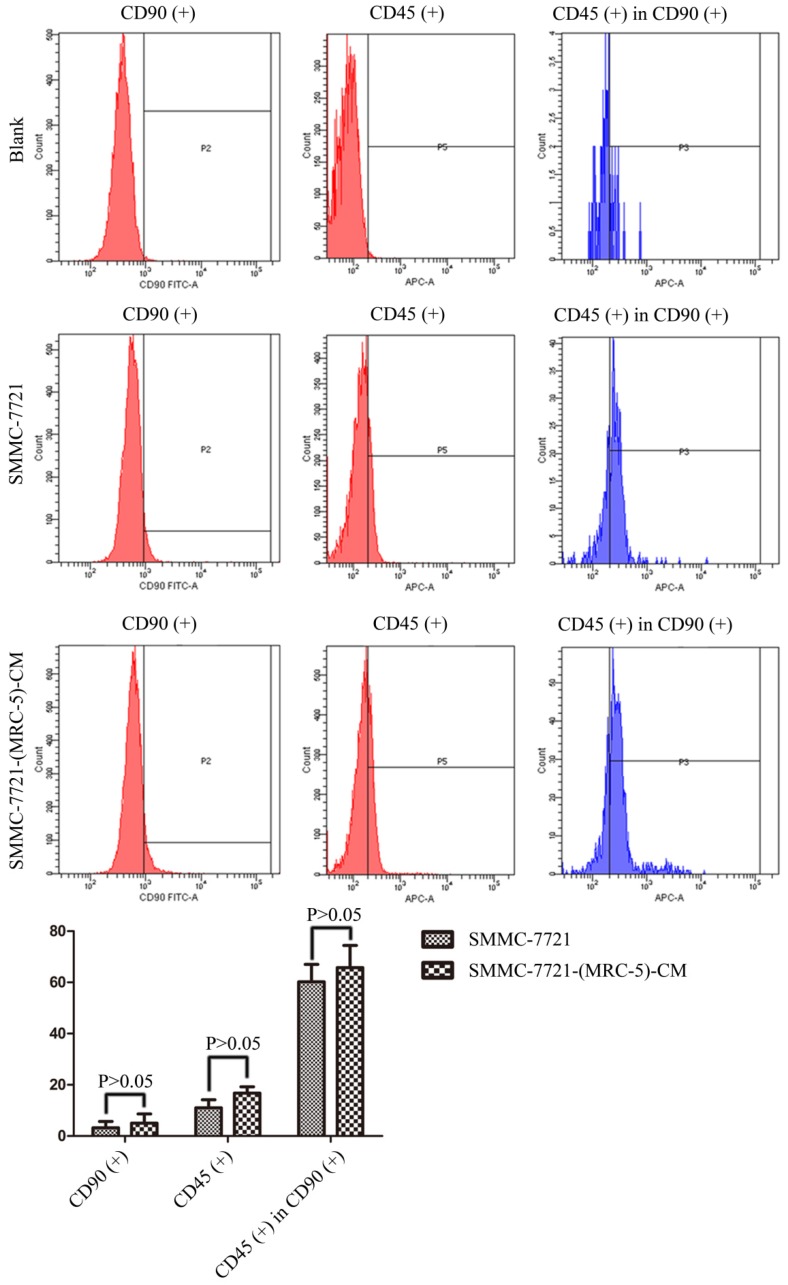
The CD90+ and CD90+/CD45- populations of SMMC-7721-(MRC-5)-CM cells compared to those of SMMC-7721 cells.

**Figure 5:**
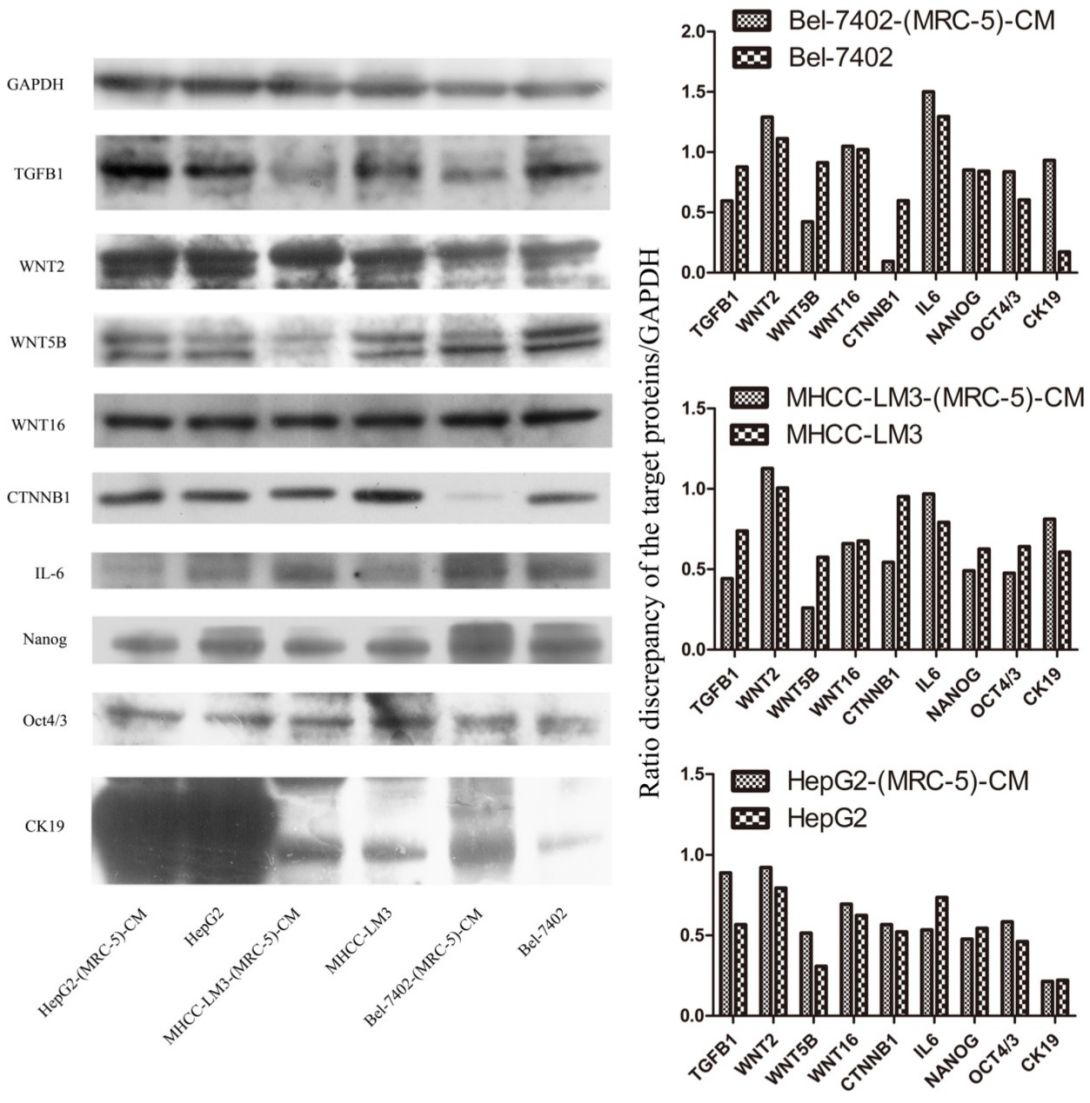
Expression of CSC markers and associated signaling molecules.

We used flow cytometry to measure the proportions of CSCs in liver cancer cells after culture in MRC-5-CM for 14 days. The proportions of CD90^+^ cells were significantly higher in MHCC-LM3-(MRC-5)-CM and HepG2-(MRC-5)-CM cells than in negative controls, and were moderately higher in Bel-7402-(MRC-5)-CM and SMMC-7721-(MRC-5)-CM cells than in negative controls (Figs. 1-4). The proportions of CD45^+^ cells were similar to those of CD90^+^ cells. However, the proportions of CD90+/CD45- cells were slightly higher in Bel-7402-(MRC-5)-CM and MHCC-LM3-(MRC-5)-CM cells than in controls, but were much lower in HepG2-(MRC-5)-CM and SMMC-7721-(MRC-5)-CM cells, compared to controls. We also evaluated the expression levels of other CSC markers, including Nanog, Oct4/3, CK19, and their associated signaling molecules, TGFB1/CTNNB1, IL6, WNT2, WNT5B, and WNT16, by Western blotting (Fig. 5). Nanog was downregulated in MHCC-LM3-(MRC-5)-CM and HepG2-(MRC-5)-CM cells; Oct4/3 was upregulated in Bel-7402-(MRC-5)-CM and HepG2-(MRC-5)-CM cells; and CK19 was upregulated in Bel-7402-(MRC-5)-CM and MHCC-LM3-(MRC-5)-CM cells. TGFB1/CTNNB1 was downregulated in Bel-7402-(MRC-5)-CM and MHCC-LM3-(MRC-5)-CM cells but upregulated in HepG2-(MRC-5)-CM cells, compared to controls. IL6 was upregulated in Bel-7402-(MRC-5)-CM and MHCC-LM3-(MRC-5)-CM cells, but downregulated in HepG2-(MRC-5)-CM cells, compared to controls. WNT-2 was upregulated in liver cancer cells cultured in MRC-5-CM. WNT5B was downregulated in Bel-7402-(MRC-5)-CM and MHCC-LM3-(MRC-5)-CM cells, but upregulated in HepG2-(MRC-5)-CM cells, compared to controls. WNT16 expression did not differ markedly between test and control cells. Altogether, the data showed that MRC-5-CM extensively affected the phenotype of liver cancer CSCs. The details were cell-line dependent.

### Effects of MRC-5 CM on the expression of inflammation-associated cell surface molecules

**Figure 6:**
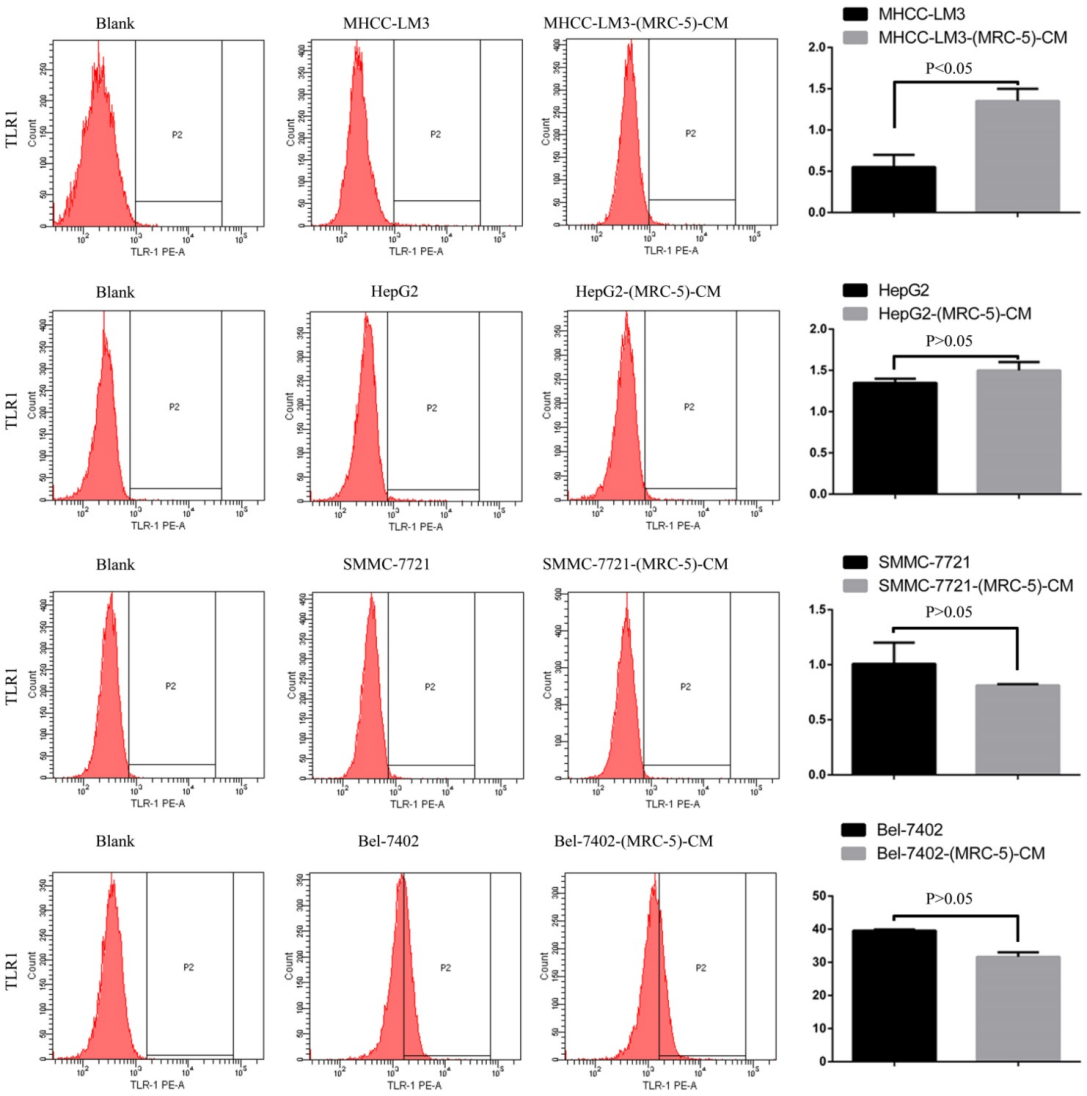
TLR1+ cells in liver cancer cells cultured in MRC-5-CM and controls.

**Figure 7:**
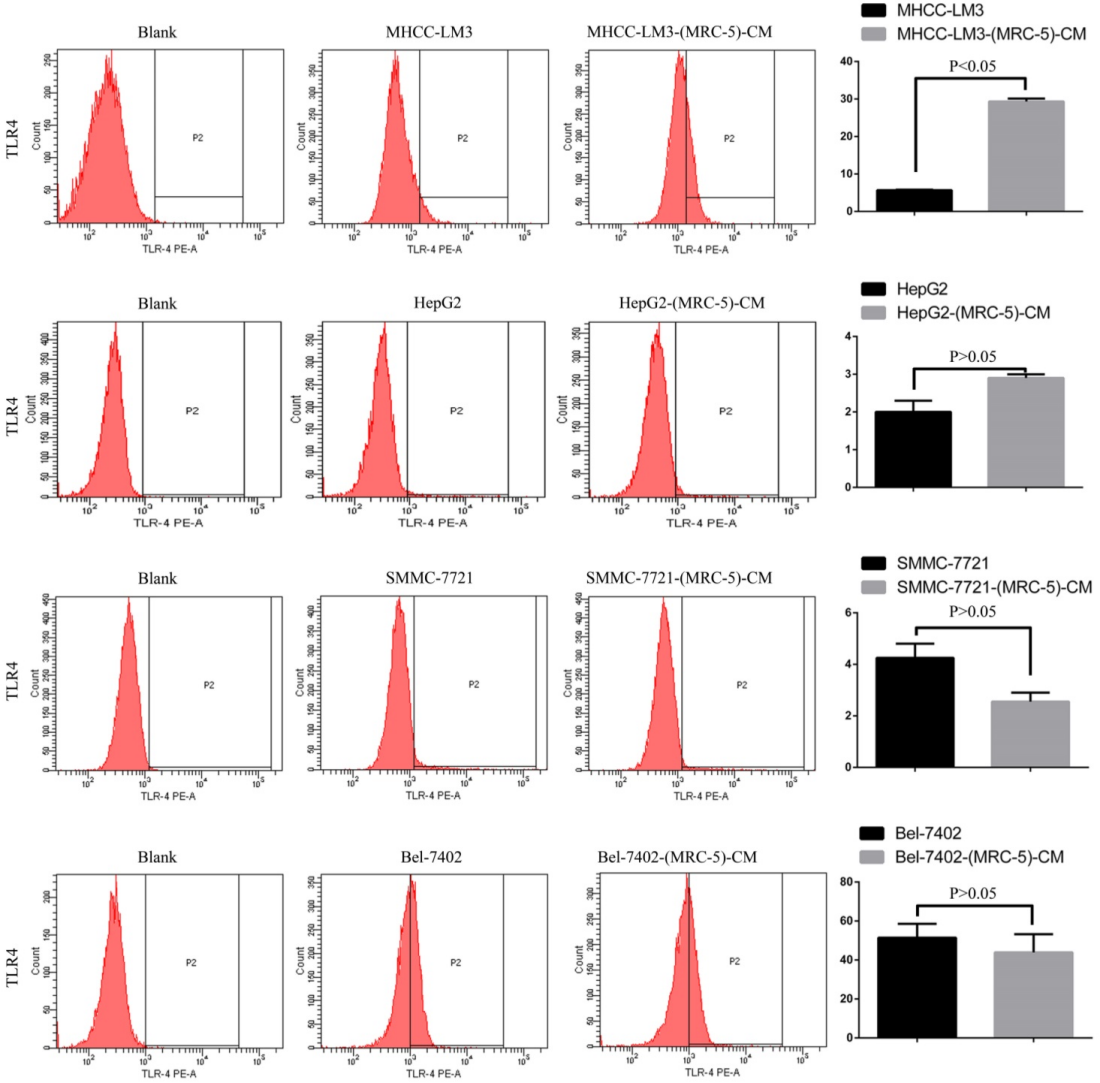
TLR4+ cells in liver cancer cells cultured in MRC-5-CM and controls.

**Figure 8:**
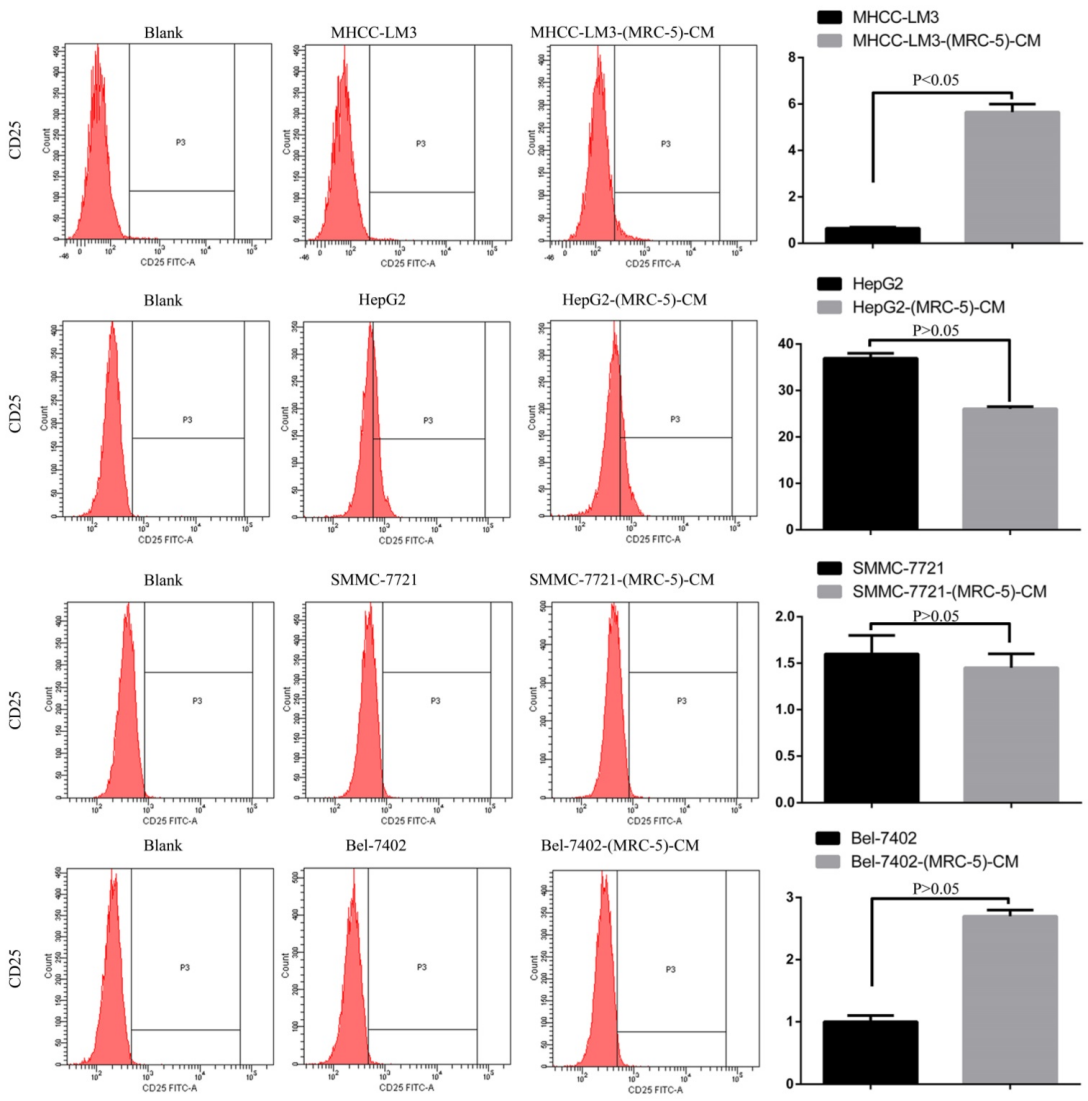
CD25+ cells in liver cancer cells cultured in MRC-5-CM and controls.

**Figure 9:**
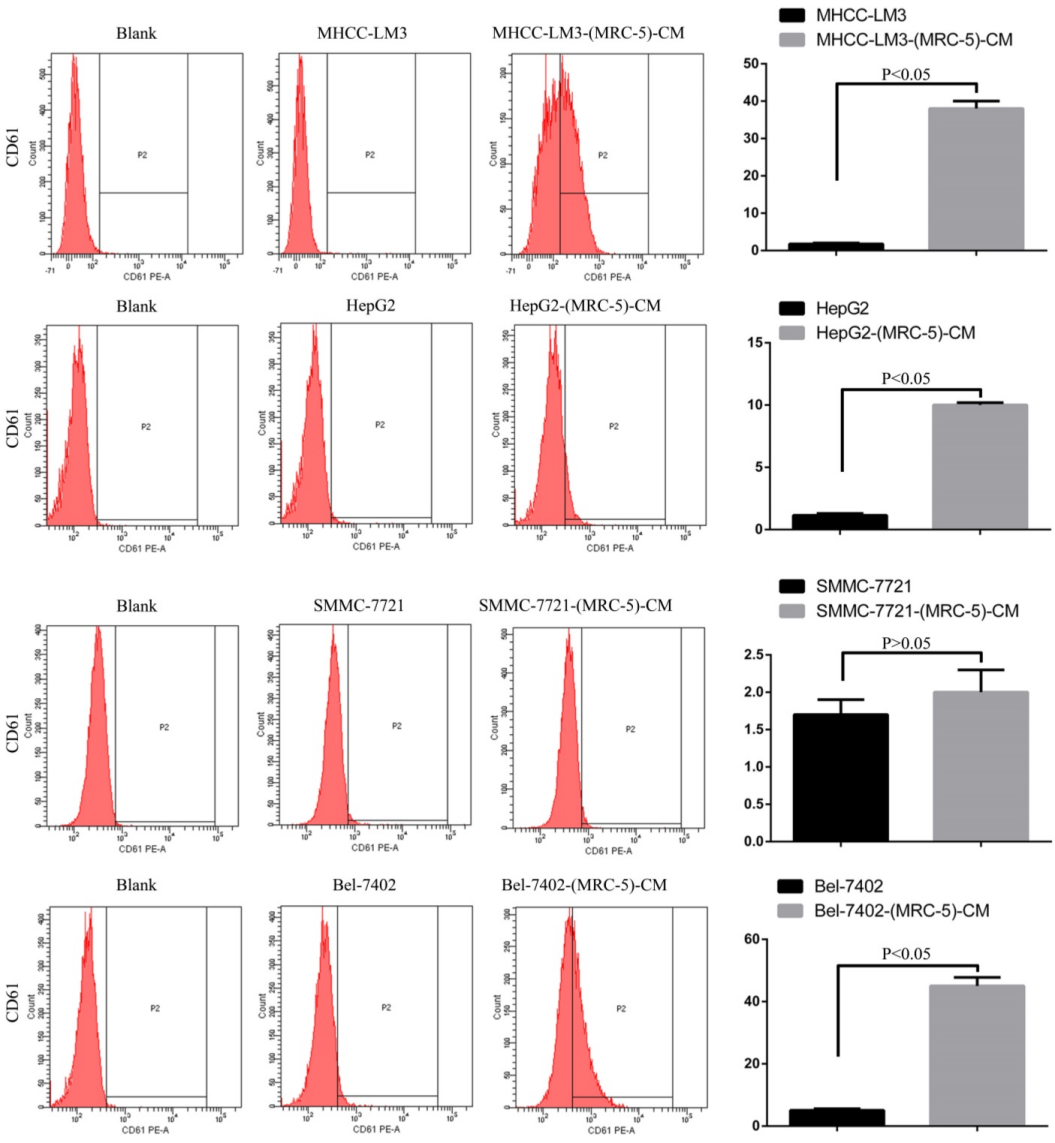
CD61+ cells in liver cancer cells cultured in MRC-5-CM and controls.

**Figure 10:**
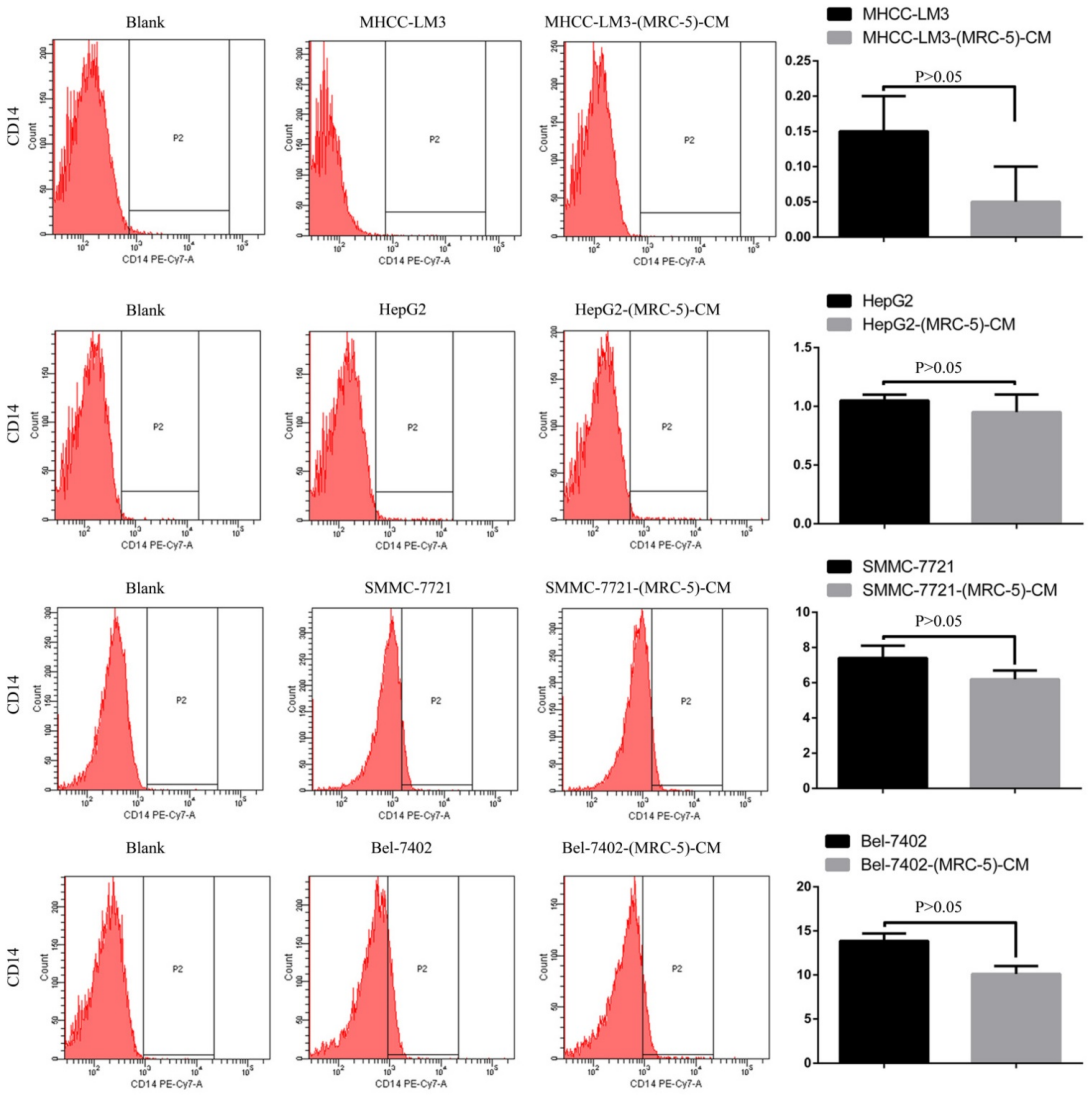
CD14+ cells in liver cancer cells cultured in MRC-5-CM and controls.

**Figure 11:**
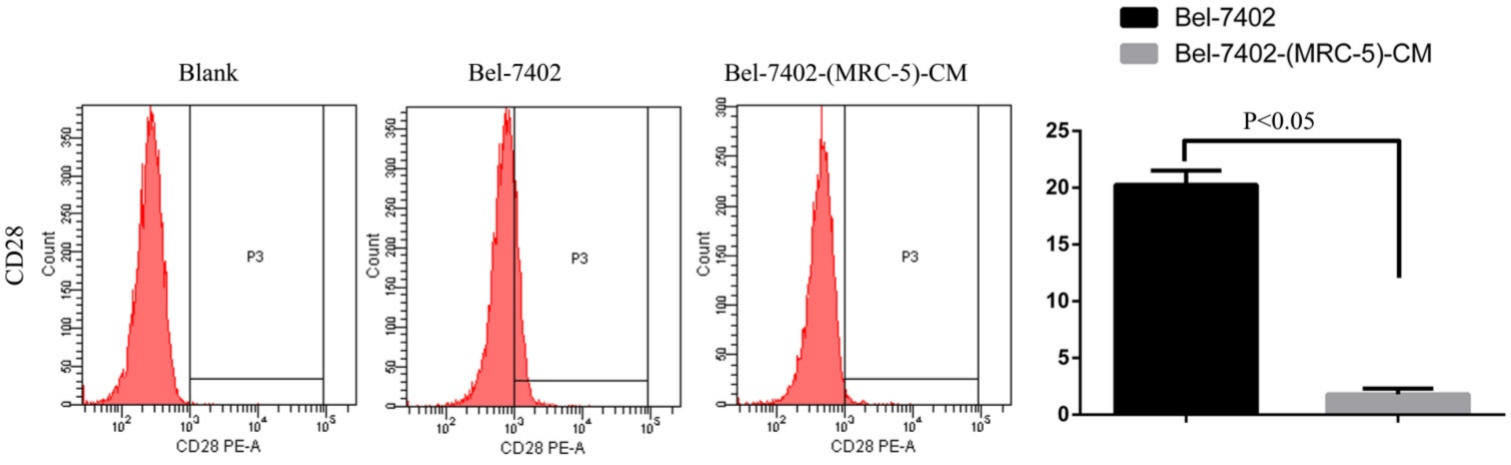
CD28+ cells in liver cancer cells cultured in MRC-5-CM and control

We used flow cytometry to explore the effects of MRC-5 CM on the expression of inflammation-associated cell surface molecules by liver cancer cells. The proportions of TLR1^+^ and TLR4^+^ cells were significantly higher in MHCC-LM3-(MRC-5)-CM cells than in controls, and were moderately higher in HepG2-(MRC-5)-CM cells relative to negative controls (Figs. 6-7). However, the proportions of cells expressing TLR1^+^ and TLR4^+^ were lower in Bel-7402-(MRC-5)-CM and SMMC-7721-(MRC-5)-CM cells than in negative controls. The proportions of cells expressing CD25^+^ were lower in HepG2-(MRC-5)-CM and SMMC-7721-(MRC-5)-CM cells than in negative controls, but higher in MHCC-LM3-(MRC-5)-CM and Bel-7402-(MRC-5)-CM cells than in negative controls (Fig. 8). The proportion of cells expressing CD61^+^ was greater in liver cancer cells cultured in MRC-5-CM than in liver cancer cells cultured in normal medium (Fig. 9). The proportion of cells expressing CD14 was lower in liver cancer cells cultured in MRC-5-CM than in liver cancer cells cultured in normal medium (Fig. 10). Nonetheless, the basic expression level of CD14 in MHCC-LM3 and HepG2 is not high. The proportion of CD28^+^ cells was significantly lower in Bel-7402-(MRC-5)-CM cells compared to negative controls (Fig. 11). These results indicate that MRC-5 modulated the expression of leukocyte differentiation antigens and TLRs in a paracrine manner.

## Discussions

Interplay between cancer cells and the TME is important during cancer evolution. It is very important to remember that such interaction is bidirectional. For example, CAFs (major players in the TME) originating from fibroblasts, myofibroblasts, and endothelial cells influence the biological behavior of cancer cells by secreting growth factors, depositing ECM, inducing angiogenic factors, and recruiting cancer- associated macrophages 17. On the other hand, CAFs can be activated by cancer cells and cancer-associated macrophages. Conceivably, CAFs account for a major contribution to tumor onset and progression, especially for liver cancer, which frequently develops against a background of chronic inflammation associated with liver cirrhosis 18. Metastatic tumor formation and growth are the final steps in solid cancer progression and are the principal cause of death 9. Undoubtedly, this process is affected by the microenvironment created in the distant metastatic organ. Therefore, it is not enough to focus on the preliminary (early) stage of tumor metastasis. On the contrary, it is important to seek to prevent the formation and growth of metastases. Such a preventive strategy is based on the notion that no matter how motile the cancer cells may be, “rude visits” should be reduced because no suitable soil is available for growth.

The lungs, which are rich in fibroblasts, are the most common site of liver cancer spread 16. We have previously shown that MRC-5 CM influences the biological behavior of liver cancer cells. For example, MRC-5 CM induced cytological changes and inhibited colony formation. In the present study, we have also observed that MRC-5 CM inhibited colony formation by SMMC-7721-(MRC-5)-CM and HepG2-(MRC-5)-CM cells (Additional Files 1: Figure S1). The expression levels of CTNNA1 and integrin β7 were lower in HepG2-(MRC-5)-CM cells than in negative controls. We hypothesize that both CSCs and cell polarity changes contribute to these alterations.

CSCs constitute only a small proportion of cancer cells, but they play a most important role in tumor formation and distant spread 19. CSCs have also been implicated in postoperative recurrence and the development of resistance to chemotherapeutic drugs 20. It is generally thought that CSCs emerge in response to tumor development. Certainly, CSCs must adapt to the TME. A permissive tumor microenvironment sustains CSC expansion, whereas a suppressive tumor microenvironment drives CSCs into “hibernation.” CSCs can switch from a dormant to an active state under appropriate conditions. Once CSCs become activated, many highly heterogeneous daughter cancer cells are generated. The detection and elimination of CSCs would seem to be an ideal method by which cancer can be defeated. Therefore, identification of CSC markers has become a major thrust of cancer research. However, how to induce CSCs to enter dormancy, or when they will “hibernate”, is not known. Fortunately, markers of liver cancer CSCs have been identified; these include CD90, CD45, CD24, CD44, CD133, CK19, CK7, SOX2, Oct3/4, and Nanog.

In this study, we found that CD90^+^ cells formed a higher proportion of liver cancer cells cultured in MRC-5-CM than that of control cells; CD90^+^/CD45^-^ cells were more common in Bel-7402-(MRC-5)-CM and MHCC-LM3-(MRC-5)-CM cells, but less common in HepG2-(MRC-5)-CM and SMMC-7721-(MRC-5)-CM cells, relative to controls. Nanog was downregulated in MHCC-LM3-(MRC-5)-CM and HepG2-(MRC-5)-CM cells compared to controls. POU5F1 (OCT4/3) was downregulated in MHCC-LM3-(MRC-5)-CM cells but upregulated in Bel-7402-(MRC-5)-CM and HepG2-(MRC-5)-CM cells, relative to controls. CK19 was upregulated in Bel-7402-(MRC-5)-CM and MHCC-LM3-(MRC-5)-CM cells compared to controls. This is attributable to the heterogeneity of CSCs. It has become increasingly evident that normal cells can become stem-like cells under certain conditions, as can cancer cells 21. In other words, MRC-5-CM extensively influenced the phenotype of liver cancer CSCs, but the details were cell-line dependent.

We also used Western blotting to evaluate the expression of TGFB1/CTNNB1, WNT2, WNT5B, WNT16, and IL6. We found that TGFB1/CTNNB1 was downregulated in Bel-7402-(MRC-5)-CM and MHCC-LM3-(MRC-5)-CM cells, but upregulated in HepG2-(MRC-5)-CM cells, compared to controls. IL6 was upregulated in Bel-7402-(MRC-5)-CM and MHCC-LM3-(MRC-5)-CM cells, but downregulated in HepG2-(MRC-5)-CM cells, relative to controls. WNT-2 was upregulated in liver cancer cells cultured in MRC-5-CM. WNT5B was downregulated in Bel-7402-(MRC-5)-CM and MHCC-LM3-(MRC-5)-CM cells, but upregulated in HepG2-(MRC-5)-CM cells, relative to controls. Such results indicate that changes in the biological behavior of tumor cells are not caused by a single factor. The complexity of the biological behavior of tumor cells seems to involve factors beyond those of tumor cells per se. We need to ask the questions: How can cancer cells exhibit such complex biological behavior? Is every step of metastasis well-orchestrated or is the process stochastic? These questions require a great deal of thought.

About 15% of human cancers are associated with chronic inflammation 22. Chronic hepatitis is not only central to liver cancer pathogenesis but also essential for progression. After liver damage occurs, hepatic macrophages secrete pro-inflammatory cytokines and chemokines, including IL-1β and TNF, activating the inflammation-tumorigenesis cascade and triggering the immune escape that fuels the development of liver cancer 17.

Macrophages also play roles in distant metastasis and the associated poor prognosis of liver cancer by secreting IL-10, TGF-β, and other chemokines 23. Notably, macrophages can be activated by both cancer cells and CAFs. Moreover, normal cells can convert to a stem-cell-like state under certain conditions. Thus, we evaluated the influence of MRC-5-CM on expression of TLRs and leukocyte-differentiation antigens (CD14, CD25, CD28, and CD61) in liver cancer cells. The proportions of TLR1^+^ and TLR4^+^ cells were higher in MHCC-LM3-(MRC-5)-CM and HepG2-(MRC-5)-CM cells than in negative controls. However, the reverse was true of Bel-7402-(MRC-5)-CM and SMMC-7721-(MRC-5)-CM cells. The proportions of CD25^+^ cells were lower in HepG2-(MRC-5)-CM and SMMC-7721-(MRC-5)-CM cells than in negative controls, but the reverse was true of MHCC-LM3-(MRC-5)-CM and Bel-7402-(MRC-5)-CM cells. The proportion of CD61^+^ cells was higher in liver cancer cells cultured in MRC-5-CM than in liver cancer cells cultured in normal medium. The proportion of CD14^+^ cells was seemingly lower in liver cancer cells cultured in MRC-5-CM than in liver cancer cells cultured in normal medium. The proportion of CD28^+^ cells was significantly lower in Bel-7402-(MRC-5)-CM cells than in negative controls. Recently, several studies have described relationships among TLRs, CD14, CD25, CD28, and CD61 and the evolution of human cancers. We found that CD14, CD25, and CD28 can also be expressed by liver cancer cells, but their roles need to be further explored. CD61^+^ (a putative marker of breast cancer CSCs) had been well-studied, but the role of CD61 in liver cancer remains unclear.

## Conclusions

Molecularly targeted therapy of the TME has become a hot topic in the field of cancer research. However, the heterogeneity of cancer cells and the complexity of the TME render it challenging to improve therapies. When molecularly targeted therapy is contemplated, it is essential to detect relevant genes to define ideal therapeutic targets. MRC-5 influences the production by liver cancer cells of CSC markers and inflammation-associated cell surface molecules. However, the details are cell-line dependent and the underlying molecular mechanisms require further research.

## Supplementary Material

Supplementary figure S1.

## References

[1] Miller KD, Siegel RL, Lin CC (2016). Cancer treatment and survivorship statistics, 2016. CA Cancer J Clin.

[2] Leonardi GC, Candido S, Cervello M (2012). The tumor microenvironment in hepatocellular carcinoma (review). Int J Oncol.

[3] Thiery JP, Acloque H, Huang RY (2009). Epithelial-mesenchymal transitions in development and disease. Cell.

[4] Polyak K, Hahn WC (2006). Roots and stems: stem cells in cancer. Nat Med.

[5] Coulouarn C, Clément B (2014). Stellate cells and the development of liver cancer: therapeutic potential of targeting the stroma. J Hepatol.

[6] Yang JD, Roberts LR (2010). Hepatocellular carcinoma: A global view. Nat Rev Gastroenterol Hepatol.

[7] Virginia Hernandez-Gea, Sara Toffanin, Scott L (2013). Friedman, et al. Role of the Microenvironment in the Pathogenesis and Treatment of Hepatocellular Carcinoma. Gastroenterology.

[8] Keigo Machida (2010). TLRs, Alcohol, HCV, and Tumorigenesis. Gastroenterol Res Pract.

[9] Iwatsuki M, Mimori K, Yokobori T (2010). Epithelial-mesenchymal transition in cancer development and its clinical significance. Cancer Sci.

[10] Fábián A, Barok M, Vereb G (2009). Die hard: are cancer stem cells the Bruce Willises of tumor biology?. Cytometry A.

[11] Ratajczak MZ (2005). Cancer stem cells-normal stem cells "Jedi" that went over to the "dark side". Folia Histochem Cytobiol.

[12] Xiang Y, Yang T, Pang BY (2016). The Progress and Prospects of Putative Biomarkers for Liver Cancer Stem Cells in Hepatocellular Carcinoma. Stem Cells Int.

[13] Yang ZF, Ho DW, Ng MN (2008). Significance of CD90+ cancer stem cells in human liver cancer. Cancer Cell.

[14] Durnez A, Verslype C, Nevens F (2006). The clinicopathological and prognostic relevance of cytokeratin 7 and 19 expression in hepatocellular carcinoma. A possible progenitor cell origin. Histopathology.

[15] Lebret SC, Newgreen DF, Thompson EW (2007). Induction of epithelial to mesenchymal transition in PMC42-LA human breast carcinoma cells by carcinoma-associated fibroblast secreted factors. Breast Cancer Res.

[16] Uka K, Aikata H, Takaki S (2007). Clinical features and prognosis of patients with extrahepatic metastases from hepatocellular carcinoma. World J Gastroenterol.

[17] Heindryckx F, Gerwins P (2015). Targeting the tumor stroma in hepatocellular carcinoma. World J Hepatol.

[18] Augusto Villanueva, Josep M (2011). Llovet. Targeted Therapies for Hepatocellular Carcinoma. Gastroenterology.

[19] Jordan CT, Guzman ML, Noble M (2006). Cancer stem cells. N Engl J Med.

[20] Ma S, Lee TK, Zheng BJ (2008). CD133+ HCC cancer stem cells confer chemoresistance by preferential expression of the Akt/PKB survival pathway. Oncogene.

[21] Chaffer CL, Brueckmann I, Scheel C (2011). Normal and neoplastic nonstem cells can spontaneously convert to a stem-like state. Proc Natl Acad Sci USA.

[22] Coussens LM, Werb Z (2002). Inflammation and cancer. Nature.

[23] Capece D, Fischietti M, Verzella D (2013). The inflammatory microenvironment in hepatocellular carcinoma: a pivotal role for tumor-associated macrophages. Biomed Res Int.

