# Supplementary material for: MRC-5 Cancer-associated Fibroblasts Influence Production of Cancer Stem Cell Markers and Inflammation-associated Cell Surface Molecules, in Liver Cancer Cell Lines
Source: Int J Med Sci. 2019 Aug 6;16(8):1157–70. doi: 10.7150/ijms.34758 (PMC6743285; doi:10.7150/ijms.34758)

Supporting legend

Fig. S1. Effect of MRC-5 CM on cell proliferation and polarity. a) The colony-formation capacity of HCC cells cultured in MRC-5-CM was lower than that of control cells. b) CTNNA1 and integrinβ7 were expressed at lower levels in HepG2-(MRC-5)-CM cells than in negative controls.

Fig. S1

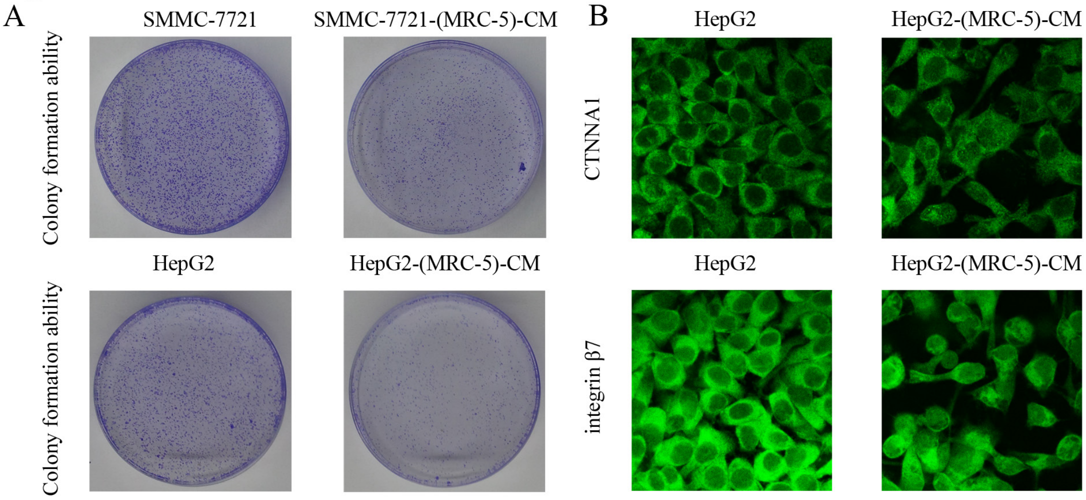

Supplement: Supplementary file 1 — Supplementary figure S1. [file ijmsv16p1157s1.pdf]
